# Well-developed spatial reversal learning abilities in harbor seals (*Phoca vitulina*)

**DOI:** 10.1007/s10071-022-01651-3

**Published:** 2022-07-16

**Authors:** Benedikt Niesterok, Shanie Martin, Lisa Hildebrand, Guido Dehnhardt, Frederike D. Hanke

**Affiliations:** 1 Institute for Biosciences, Sensory and Cognitive Ecology, University of Rostock, Albert-Einstein-Str. 3, 18059 Rostock, Germany; 2 Faculty of Science and Engineering, Biological and Marine Science, University of Plymouth, Drake Circus, Plymouth, PL4 8AA UK; 3 Geospatial Ecology of Marine Megafauna Lab, Marine Mammal Institute, and Department of Fisheries, Wildlife and Conservation Sciences, Oregon State University, Newport, OR 97365 USA; 4 Institute for Bioscience, Neuroethology, University of Rostock, Albert-Einstein-Str. 3, 18059 Rostock, Germany

**Keywords:** Conditional reversal learning, Behavioral flexibility, Within session reversal, Cognition, Pinnipeds

## Abstract

In this study, behavioral plasticity in harbor seals was investigated in spatial reversal learning tasks of varying complexities. We started with a classic spatial reversal learning experiment with no more than one reversal per day. The seals quickly learned the task and showed progressive improvement over reversals, one seal even reaching one-trial performance. In a second approach, one seal could complete multiple reversals occurring within a session. Again, a number of reversals were finished with only one error occurring at the beginning of a session as in experiment 1 which provides evidence that the seal adopted a strategy. In a final approach, reversals within a session were marked by an external cue. This way, an errorless performance of the experimental animal was achieved in up to three consecutive reversals. In conclusion, harbor seals master spatial, in contrast to visual, reversal learning experiments with ease. The underlying behavioral flexibility can help to optimize behaviors in fluctuating or changing environments.

## Introduction

Reversal learning (RL; Pavlov 1927) involves the discrimination of two stimuli by an individual. One stimulus is associated with a reward (positive stimulus, S+), while the other stimulus is associated with no reward (negative stimulus, S−). These relations have to be learned during the acquisition phase until the learning criterion is reached. Then, going beyond mere discrimination learning, the reward stimuli contingencies are reversed, which is defined as a reversal (R). Consequently, the prior S+ becomes the new S- and vice versa. In a serial reversal learning (SRL) experiment, this procedure is repeated over and over again, every time the animal meets the learning criterion. If the animal learns to learn (Harlow 1949) over a series of Rs, the number of errors to the preset criterion decreases over time, and the animal can/might be able to reach the ultimate performance, one-trial learning. One-trial learning is defined as an errorless performance following a single error at the beginning of a new R. If one-trial learning occurs, the animal has formed a RL set. Thus, it has learned to learn (Harlow 1949) most likely by developing rules according to which it can solve new discrimination problems within a single trial. Such a rule could be a “win-stay, lose-shift” strategy (Levine 1959). In contrast, without the formation of a learning set, the animal needs to learn how to respond appropriately every time the conditions change which requires more time. With respect to RL, this would mean that an individual has to “re-learn” the stimulus reward contingencies in every new R.

The formation of a RL set allows animals to efficiently and rapidly respond to changing conditions in their environment and might be crucial for survival. Thus, RL is considered to be a measure of behavioral flexibility. Its adaptive value has been discussed regarding opposing or fluctuating ecological contexts such as when an organism is confronted with fluctuating resources (Davey 1989; Day et al. 1999; Shettleworth 1998), complex or fast changing environments (Jones 2005; Robinson 1985) as well as complex social structures (Bhumstein and Armitage 1998; Easton 2005; Shultz and Dunbar 2006).

So far, RL performance has been documented in numerous species including chimpanzees (*Pan troglodytes*) (Schusterman 1964), rats (*Rattus norvegicus*) (Bushnell and Stanton 1991; Dufort et al. 1954; Mackintosh and Cauty 1971), horses (*Equus ferus*) (Potter and Fiske 1979), dogs (*Canis familiaris*) (Tapp et al. 2003), pigeons (*Columba livia)* (Durlach and Mackintosh 1986; Gonzalez et al. 1967; Ploog and Williams 2010), other bird species and some reptiles (Bond et al. 2007; Day et al. 1999; Holmes and Bitterman 1966), bumblebees (*Bombus* sp.) (Chittka 1998) and cockroaches (*Periplaneta americana*) (Balderrama 1980). All these species showed progressive improvement during R training, and some species, such as chimpanzees, rats and cockroaches, even accomplished one-trial learning.

The ability to reverse has also been documented in aquatic animals. RL was examined in the common octopus (*Octopus vulgaris*) (Bublitz et al. 2017, 2021 and references therein) and in fish (Kuroda et al. 2017; Lucon-Xiccato and Bisazza 2017). Another set of RL experiments (Beach et al. 1974; Schusterman 1966; Walsh et al. 2007) focused on marine mammals, the group of animal species to which the experimental subjects of the study at hand belong to. Those studies demonstrated the ability of certain marine mammal species to reverse spatial (Beach et al. 1974), but also visual tasks (Kuroda et al. 2017; Walsh et al. 2007) successfully.

In contrast to the studies on RL in marine mammals mentioned above, a study by Erdsack et al. (this volume) on visual reversal learning in harbor seals *(Phoca vitulina)* could only demonstrate a slow, non-gradual progression of improvement over Rs and only in one out of four seals; all other seals failed to meet criterion after extensive training at various early stages within the experiment. This finding was very surprising as harbor seals are known to possess well-developed cognitive abilities allowing them to, for example, form concepts (Mauck and Dehnhardt 2005; Scholtyssek et al. 2013).

The goal of the current experiment, as a follow-up experiment to visual RL, was to contrast the harbor seals’ performance in a visual RL experiment (Erdsack et al., this volume) with their performance in a spatial RL experiment. We hypothesized that harbor seals perform better in a spatial RL experiment as previous evidence obtained in phocid seals supports the importance of spatial over visual information. First, Renouf and Gabarko (1989) tested harbor seals in a spatial and in a visual discrimination task, and all experimental animals learned the spatial discrimination task faster. Second, a harbor seal performed better in a matching-task with stimuli in landscape condition, meaning the stimuli were spaced out in the basin, in comparison to local feature presentation with the stimuli next to each other (Mauck and Dehnhardt 2007). Lastly, even in the context of RL, harp seals were able to solve a visual RL task, however, only when they were allowed to use a location shift as a conditional cue; without spatial cues, harp seals in the same study experienced difficulties completing the visual task (Walsh et al. 2007).

In this study, we trained two seals in a classic spatial RL experiment (experiment 1) that was designed to allow meaningful comparison with the previous visual RL experiment (Erdsack et al. this volume). In both experiments, the seals left a hoop station, swam toward the stimuli from a distance and responded directly at/below the stimuli. In the visual RL experiment, the seals needed to discriminate between a horizontal and vertical bar displayed in two openings of a board, whereas in the spatial RL experiment, the seals responded to one of two small boards installed to the left or right from the midline intersecting the station in which the seal was resting in the intertrial interval. As we were able to successfully train two seals in a classic spatial RL experiment (experiment 1), we analyzed the spatial RL abilities of one of the two seals in detail by performing two additional experiments. In these experiments, we confronted the seal with within-session Rs (experiment 2) and with a conditional cue indicating a R which would allow for errorless R performance (experiment 3).

## Material and methods

### Experimental animals

Two male harbor seals (Sam, 22 years old; Moe, 10 years old) were involved in the experiments. Seal Moe took part in all experiments, whereas seal Sam only participated in experiment 1 due to reasons unrelated to this study. Seal Moe had previous experimental experience in hydrodynamic tasks (Krüger et al. 2018), whereas seal Sam had already participated in numerous experiments with stimuli of many different modalities (see for example Bodson et al. 2007; Hanke and Dehnhardt 2009). Both seals had participated in the visual RL experiment (Erdsack et al. this volume). Experiments were conducted 5 days a week. Both seals were fed 2–3 kg of fish during experiments with the food amount being individually adjusted depending on weight and season. They received the food mainly during experiments but also during other daily training routines.

The experiments carried out in this study were in accordance with the European Communities Council Directive of September 22, 2010, (2010/63/EU) and the German Animal Welfare Act of 2006. The individuals used in the study were not subject to pain, suffering or injury; therefore, no approval or notification was required (Staatliches Amt für Natur und Umwelt Rostock, Landesamt für Landwirtschaft, Lebensmittelsicherheit und Fischerei, Mecklenburg-Vorpommern).

### Experimental setup

**Fig. 1:**
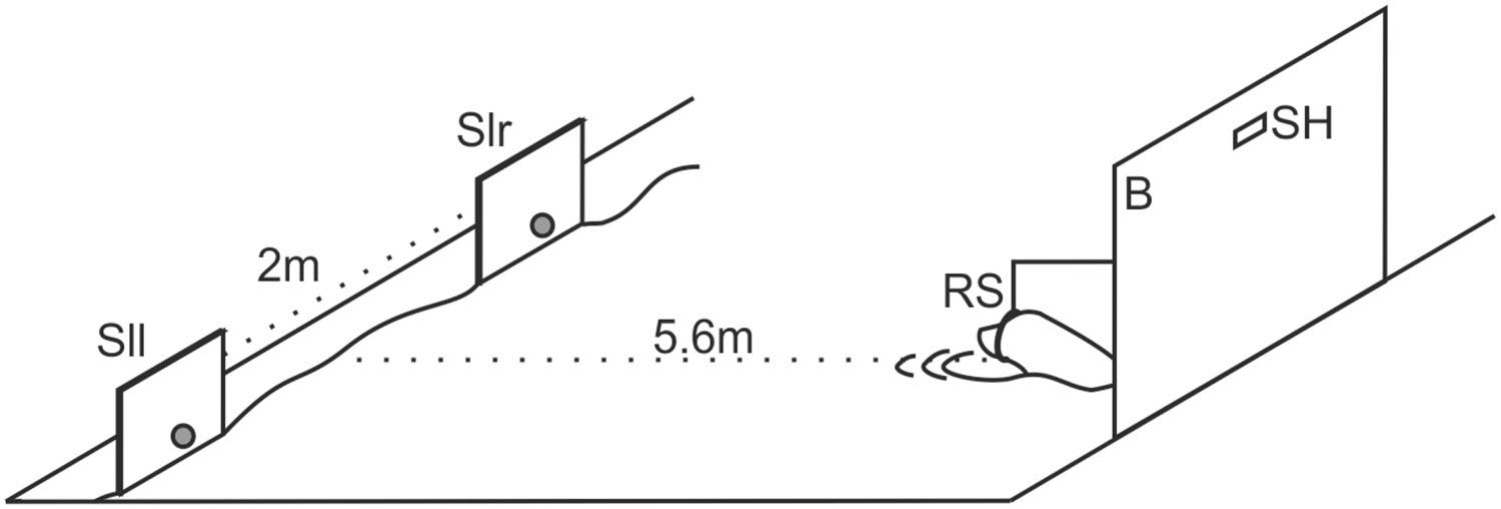
Experimental setup. At the beginning of every trial, the animal was stationing in a ring station (RS) attached to a large board (B). This board served to hide the experimenter during experiments. A spy hole (SH) within the board allowed the experimenter to oversee the actions taken place in front of the board. The two locations in space (Sl) to which the animal responded to in the course of the experiment were mounted on the opposite side of the basin to the left (Sll) and right side (Slr) from the point of view of the experimental animal

Experiments were conducted inside a netting enclosure in the water. For the spatial RL task, two identical white PVC squares (30 cm × 30 cm) were used, each having a target ball at the lower edge (Fig. 1). The two squares were suspended on one side of the experimental basin with the target balls slightly above the water surface. The distance between the two squares was 2 m. Both squares represented two spots in space; one on the right, the other on the left side of the animal. The animal was supposed to approach these squares and put its snout on the target ball to make a response. We will refer to the squares as “locations” from now on.

On the opposite side of the basin and thus opposite of the two locations, there was a PVC board (1.5 m × 1.5 m) with a small spy hole through which the experimenter could see the experimental animal. The PVC board was at 5.6 m distance to the center between both locations and served to hide the experimenter. To the front of the board, a ring station was attached by a metal arm. The ring station was mounted just above the water surface and served to position the animal facing the two locations in the intertrial interval.

### Experimental procedure and analysis

#### General

The general experimental procedure for the three experiments examining the seals’ spatial RL abilities involved that the animals discriminated between two locations marked by the two white PVC squares installed to the right and the left from the animal’s point of view. In every single trial, the animal had to first put its head through the ring station. Then, a short whistle signaled the animal to leave the ring station and make a decision. A decision was defined as touching a target ball at one of the locations. A correct decision was indicated by a long whistle upon which the animal returned to the experimenter and was rewarded with fish. In case of a wrong decision, the experimenter called “nein” (German word for “no”), and the animal remained unrewarded. After providing the feedback, the animal was required to station in the ring station again.

Statistical analyses for all of the experiments were run in R 3.3.3 (The R Foundation for Statistical Computing, Vienna, Austria).

#### Experiment 1: spatial reversal learning

In the first experiment, the harbor seals’ performance was tested in a spatial SRL task to determine whether progressive improvement occurred over a series of Rs.

Initially, a single trial was conducted as a preference test, in which the animals were allowed to choose either the right or the left location spontaneously. The location chosen during this trial was declared as S− in the acquisition phase (R0). The non-preferred location was defined as S+; for both animals, the right location was the S+ during R0. This way, the animals were not rewarded for a pre-existing preference, but instead had to learn which of the two locations was defined as S+ during R0.

During R0, the animals were trained on the original discrimination. Throughout the experiment, one session was conducted per day. One session consisted of 30 trials. Both seals had to meet the learning criterion of ≥ 80% correct choices in one session (same learning criterion as in numerous other RL studies including the visual RL experiment; Erdsack et al. this volume) before a new R was started. At the start of a R, the S- of the just finished R was redefined as the new S+. As we conducted a SRL experiment, we repeated this procedure every time the animal reached the learning criterion.

The number of errors over each reversal was analyzed, and this number of errors made over the course of one reversal was compared between individuals. Additionally, the first trials of all sessions were analyzed to assess if the animal remembered the S+ from the previous day.

#### Experiment 2: spatial within-session reversal learning

In the second experiment, it was tested whether a harbor seal is able to reverse a spatial discrimination task even faster by performing several Rs within a single session.

In this experiment, 2–5 Rs were run within a session; a session consisted of 27–60 trials. A new R was started if the animal achieved a predefined number of correct choices in a row without errors in between. According to statistics, five correct trials in a row are needed at minimum to confirm that the animal chose the S+ with a probability significantly higher than chance (right-sided binomial test). We asked the seal to perform more correct choices, 10–25 correct choices, in a row for a stricter criterion. The number of trials to reach criterion was pre-determined by chance. We did not reverse after a fixed number of correct choices to avoid that the animal could determine the point of reversal by taking factors such as the number of trials or time into account (also holds for experiment 3). If, during the course of a R, the animal made an error, the correct trials performed so far expired; the animal had to restart to meet the required number of correct trials. Eleven Rs were started at the end of a session and continued at the beginning of the next session, to control that the animal could not use the start of a new session as a trigger for the next R; if a reversal had always been finished at the end of a session, the seal could have learnt that with every new session, it needs to choose the former S- to get a reward directly.

As in experiment 1, we analyzed the number of errors per reversal and conducted a first trial analysis.

#### Experiment 3: spatial conditional reversal learning

In the third experiment, it was investigated whether a harbor seal is not only able to reverse a spatial discrimination task quickly, but also to do this without error with the help of a conditional cue marking the beginning of a new R.

The conditional cue was an acoustic cue, the ring of a bike bell. At the beginning of a session, the S+ was defined as the location that had been the S− in the last completed R. This implies that the S+ was the same as in the final phase of the previous session, if the R had not been completed during the previous session. However, the S+ was changed if the R had been completed in the previous session. The following Rs were introduced by the sound of the bike bell. In phase 1, it was rung before the start whistle, while in phase 2, it was rung twice after the start whistle. This change in experimental procedure became necessary as the animal did not learn the conditional cue in phase 1.

In both phases, between two to five Rs were run within a session. The criterion to fulfill a R varied between 5 and 14 correct trials in a row, the number being predetermined before the onset of the session by chance, with 5 correct trials in succession being the statistical minimum as described for Experiment 2. We analyzed the proportion of correct shifts in blocks of ten reversals over time. This analysis was done separately for phase 1 and phase 2. The last 4 blocks of Rs (38 Rs) containing a high number of correct shifts were chosen to analyze the number of successful Rs defined by a correct shift and a minimum of 5 correct choices after the shift.

## Results

### Experiment 1: spatial reversal learning

**Fig. 2:**
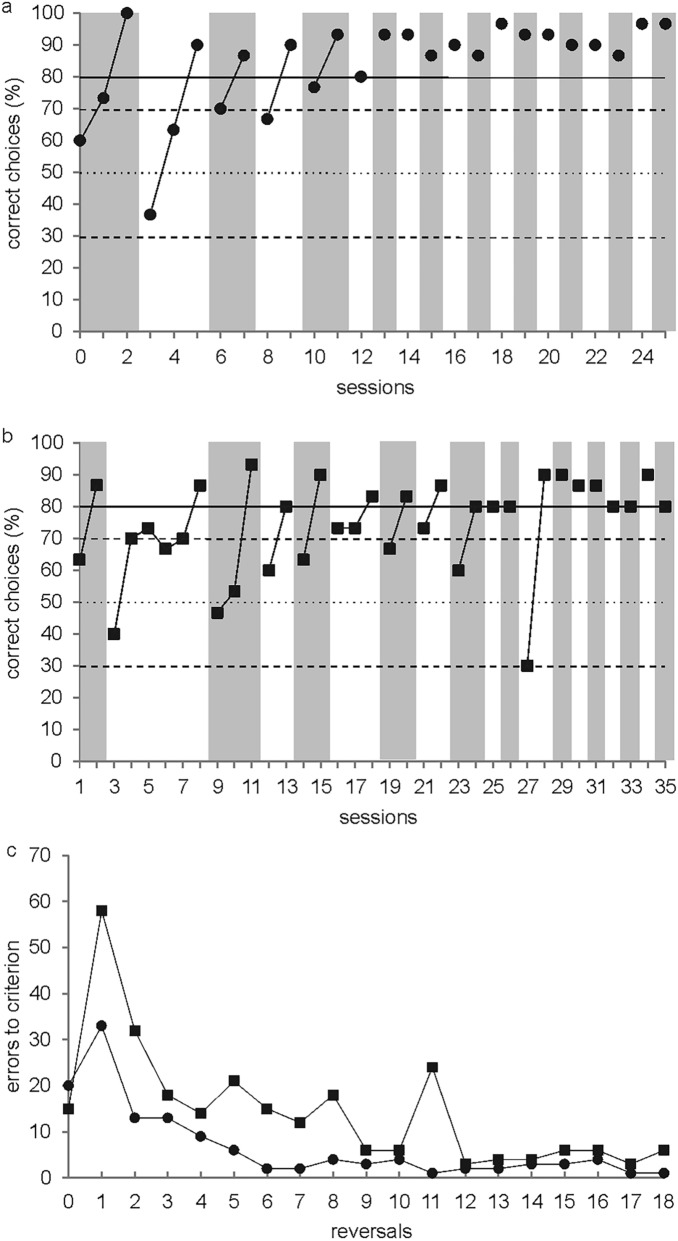
Results of serial spatial reversal learning (experiment 1). Performance of **a** seal Moe and **b** seal Sam plotted per session. The horizontal solid line represents a performance of 80% correct choices which needed to be met or surpassed in one session to meet the learning criterion. The horizontal dotted line marks chance performance at 50% correct choices, and the two horizontal dashed lines the upper and lower significance level (*p* = 0.05). The grey vertical bars indicate the performance of the seals within a single reversal. **c** Error rate for seal Moe (filled circles) and seal Sam (filled squares) plotting the number of errors as a function of the reversals

Both harbor seals learned the initial spatial discrimination task in R0. Seal Moe reached the learning criterion in R0 after three sessions (Fig. 2a), seal Sam within two sessions (Fig. 2b). The error rate plotting the number of errors for each R and animal is given in Fig. 2c. For both animals, the error rate increased from R0 to R1, but significantly decreased from R1 to R18 (*F*-statistic: *F* = 17.98 for seal Sam, *F* = 13.18 for seal Moe; *p* < 0.01). Both harbor seals, therefore, showed progressive improvement over a series of Rs. The last R of experiment 1 was completed faster than R0. Seal Moe even performed R11, R17, and R18 with a single error only; in R17, this error occurred in the first trial as expected during one-trial learning. In general, seal Moe made significantly fewer errors than seal Sam (paired *t* test *t* = 4.44, *df* = 17, *p* < 0.001).

A first trial analysis was run for both seals to find out whether the S+ from the previous session was remembered the next session; irrespective of whether a R had been completed at the end of the previous session or not. Both seals significantly continued choosing the S+ from the previous session. Seal Sam chose the previous S+ in 23 out of 35 cases (one-sided binomial test; *p* = 0.045), seal Moe in 18 out of 26 cases (one-sided binomial test; *p* = 0.038).

During the final phase of experiment 1, the animals needed only one session to complete a R resulting in one successful R per day. We separately analyzed the first trials of these Rs to find out whether the experimental animals still kept choosing the S+ from the previous day, when the S+ was switched daily; it was worthwhile to see if the animals switched autonomously to the new S+ at the onset of the R the day after, therefore using a new day as a conditional cue. Seal Moe successfully solved the last 15 Rs on a daily basis, seal Sam the last 8 Rs. Apparently both seals did not always use a new day as a trigger to autonomously switch to the new S+ . Seal Moe kept choosing the S+ from the previous day in 10 out of 15 cases (one-sided binomial test; *p* = 0.15), seal Sam remained on the side of the previous S + in the first trial of a new session in four out of 8 Rs (one-sided binomial test; *p* = 0.64).

### Experiment 2: spatial within-session reversal learning

Harbor seal Moe was able to successfully reverse a spatial discrimination task a few times within a session. Altogether 53 Rs were completed in 20 sessions.

**Fig. 3:**
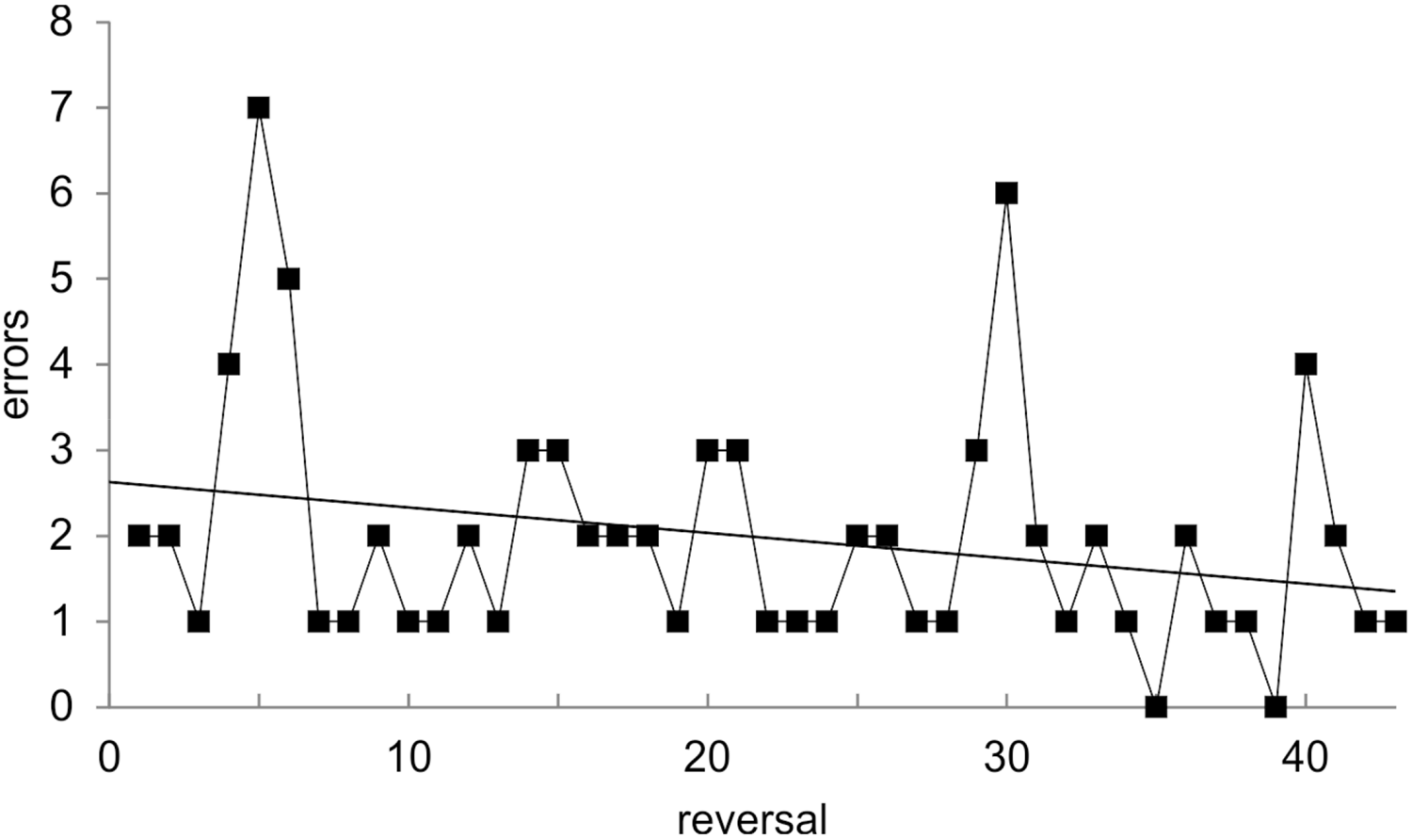
Results of spatial within-session reversal learning (experiment 2). Error rate over all within-session reversals; black solid line represents the linear regression model

In 38 out of the 53 Rs (one sided binomial test; *p* = 0.011), the performance within one R was at least 80% correct, demonstrating that the animal was working on a high performance level. The absolute number of errors for all Rs, excluding those that were conducted over two sessions, is given in Fig. 3; Rs over two sessions were excluded from error analysis because they were not within-session Rs. The absolute number of errors in the within-session Rs ranged from one to seven and decreased by tendency over Rs. However, this tendency did not turn out to be significant (*F*-statistic; *p* = 0.097; *R*^2^ = 0.043). In 18 Rs, the animal performed only a single error (one-error Rs; Fig. 3). For all these 18 one-error Rs, the error occurred right at the beginning of the R. Therefore, the experience of a single incorrect trial was sufficient for the animal to reverse its behavior in line with one-trial learning. In another 13 Rs, the animal performed two errors, and more than two errors occurred in 10 Rs. Two Rs were performed by the animal with zero errors. In these two cases, the animal accidentally responded correctly in the first trial and then continued responding to the S+ due to the positive feedback.

As in Experiment 1, it was analyzed whether the animal remembered the S+ from the last R of the previous session. Thus, each final S+ of a session and its following first choice in a new session were included in this analysis. Remembering the last S+ from the final R of the previous session was considered more challenging for the experimental animal in experiment 2 in comparison with experiment 1 because several changes of the S+ occurred within a session, and the S+ changed far more often than during experiment 1. A first trial analysis revealed that the seal only started a new session with the stimulus which had been the S+ in the last R of the previous session in 6 out of 19 cases. This result is not statistically significant (one-sided binomial test; *p* = 0.99). Thus, the animal did not seem to remember the last S+ from the previous session. Instead, in the first trials of all sessions during experiment 2, the animal significantly responded on the left side (one-sided binomial test; 16 out of 19 cases, *p* = 0.002), which is rather indicative of a behavioral strategy to find out about the stimulus-reward contingencies in the particular session.

### Experiment 3: spatial conditional reversal learning

Harbor seal Moe was able to reverse a spatial discrimination task without errors if the point of reversal was linked with a conditional cue.

**Fig. 4:**
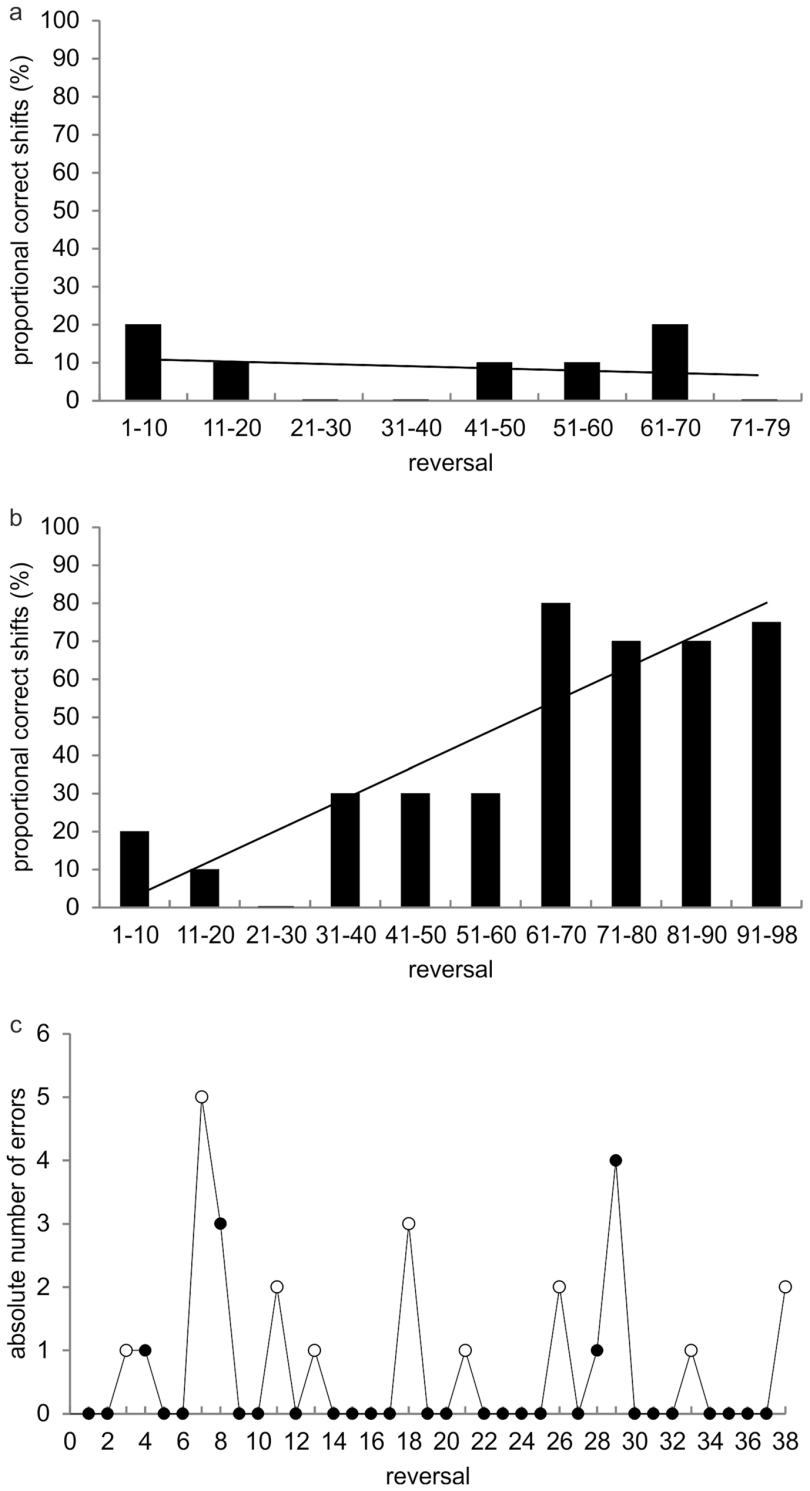
Results of conditional spatial reversal learning (experiment 3). **a** Frequency of correct shifts to the conditional cue in phase 1 of experiment 3; the 78 reversals conducted are grouped in blocks of ten, last block contains only nine reversals; black line shows regression line. **b** Frequency of correct shifts to the conditional cue in phase 2 of experiment 3; the 98 reversals are grouped in blocks of ten, last block contains only eight reversals; black line shows regression line. **c** Number of errors over the last 38 Rs; filled circles represent successful reversals with both criteria met: correct shift to the new S+ with the conditional cue and at least five correct trials in a row; open circles represent reversals during which one of the criteria was not met

However, first the conditional cue was presented once before the starting signal (phase 1), and the animal did not learn the meaning of the conditional cue. Figure 4a shows the absolute number of shifts to the S+ in response to the acoustic cue in phase 1. Rs are grouped in blocks of ten; please note that the last block of Rs comprises nine Rs only. Thus, the number of correct shifts is given as percentage of ten and nine Rs, respectively. A linear regression model was calculated for the number of correct shifts over the block of Rs. The linear model is not statistically significant (*F*-statistics; *p* = 0.71), indicating that the animal did not learn the meaning of the conditional cue in phase 1 of experiment 3.

In phase 2, the external cue was presented twice after the starting signal. This methodological modification caused an increasing number of correct shifts (*F*-statistics; *p* = 0.002) as a response to the conditional cue over Rs (Fig. 4b). Again, Rs are grouped in blocks of ten except for the last bar containing eight Rs only. The number of correct shifts is given as percentage of ten and eight Rs, respectively. Between R-block six and seven, there is an increase from 30 to 80% correct shifts. This increase strongly indicates that the animal has learned the meaning of the external cue over Rs. The number of correct shifts for R-blocks 7 to 10 (28 out of 38) is highly significant (one-sided binomial test; *p* value = 0.0025). In conclusion, the experimental animal was able to successfully switch to a new S+ in response to a conditional cue.

It was the intention of experiment 3 to test if errorless reversal learning occurs through the introduction of a conditional cue. Errorless reversal learning requires a shift to the new S+ after the conditional cue was presented as well as at least five correct responses in a row thereafter (see “Material and methods”). Therefore, the error rates of the blocks of Rs with a high percentage of correct behavioral shifts, blocks seven to ten with 38 Rs altogether, were analyzed in further detail (Fig. 4c). In 28 of these Rs, the animal significantly responded to the conditional cue and chose this new S+ for at least five times in a row (one-sided binomial test; *p* value = 0.003), but made errors in three Rs after five correct responses. In 25 Rs of the 38Rs, the animal significantly performed without errors (one-sided binomial test; *p* = 0.036). In the remaining Rs, the animal either did not switch to the new S+ after the external cue had been presented or it did not keep choosing the new S+ for at least five times in succession. In addition to the high number of errorless Rs, in two instances (Rs 30–32 and Rs 34–36), the animal was able to perform three errorless Rs in a row. This result further confirms the ability to perform successfully in a conditional within-session RL experiment.

## Discussion

In this study, we showed that two harbor seals were able to reverse a spatial task and to progressively improve their performance with one seal reaching down to one-trial learning and to errorless performance in a conditional RL experiment. We report the results from two harbor seal individuals that behave normally and showed similar results in experiment 1 (see detailed discussion below). If the behavioral flexibility underlying RL documented in these two individuals was shown by harbor seals in general, it would allow seals to adapt to changes in their complex and fluctuating environment quickly in line with an argumentation by Bond et al. (2007). Thereby the need for relearning the reward contingencies in every new situation is eliminated which is time-saving and thus crucial for survival under certain circumstances.

It is hard to imagine in which natural situation RL might explicitly be advantageous for seals; one scenario could be that offshore feeding grounds become depleted but are repopulated by prey after some time, and the seal adapts to these changes. Alternatively, RL might not be directly linked to a natural situation but harbor seals might simply possess the information processing capacity allowing them to successfully reverse (Mauck and Dehnhardt 2005), and, of course, the previous experiences as experimental animals have probably rendered our experimental animals highly adjustable to changing experimental conditions (Harlow 1949).

One of our goals was to contrast the harbor seals’ performances in a visual (Erdsack et al. this volume) with a spatial RL experiment. Comparing the results of these studies, it is obvious that our harbor seals performed better with the spatial task: (1) the harbor seals in this study performed 20 (seal Moe) and 15 errors (seal Sam), respectively, during the acquisition of the spatial discrimination task (R0). In visual RL, the best performing harbor seal learned the initial discrimination task only after 108 errors, the three other seals needed far more errors to complete R0 with the seals participating in the spatial RL experiment requiring 1343 errors (seal Moe), and 340 errors (seal Sam) in R0. (2) Both harbor seals showed progressive improvement in reversing the spatial task with one of the harbor seals performing only a single error in a few Rs. In contrast, seal Sam improved from R1 (71 sessions) to R2 (44 sessions) in the visual RL experiment, however failed to complete R3 within far more sessions (80 sessions) than in R2. Seal Moe did not master R1 within 120 sessions when working with the visual stimuli. The best performing seal in the visual RL showed non-gradual progressive improvement but only reached a minimum of six errors in R33. (3) In the current spatial RL experiment, seal Moe even mastered within-session reversals and learned to reverse upon a conditional cue. Discrepancy between the outcome of spatial and visual RL tasks have also been found in other RL studies. A number of species perform better in visual RL such as minks (*Mustela vison*) and ferrets (*Mustela furo*) (Doty and Combs 1969), or rhesus monkeys (*Macaca mulatta*) (Warren 1966). In contrast, the performance of skunks (*Mephitis mephitis*) (Doty and Combs 1969), turtles (*Chyrsemys picta*) (Holmes and Bitterman 1966), pigeons (Bullock and Bitterman 1962; Weyant 1966) and cats (Warren 1966) with spatial RL tasks is superior to visual RL tasks. A superior spatial performance, as found in our RL study with harbor seals but also with other marine mammal species (Beach et al. 1974), can be explained as follows: First, a spatial task does not include irrelevant cues, whereas in a visual task position is an irrelevant cue. The presence of an irrelevant cue might render a task more difficult to learn. Second, orientation in space and thus spatial information in general is crucial for navigating in the open ocean from and to haul-out places or foraging grounds over large distances (Thompson and Miller 1990). As a consequence, harbor seals as active foragers might demonstrate increased spatial awareness in line with previous reports (Beach et al. 1974; Day et al. 1999; Fagan et al. 2013). In contrast, visual information, although available, might often be absent or impaired due to for example turbidity, dim light conditions or even darkness. The importance of spatial information for a harbor seal is supported by previous experimental evidence (Mauck and Dehnhardt 2007; Renouf and Gaborko 1989). Thus, most likely, spatial information is more salient to harbor seals than visual information explaining the different experimental outcomes of our RL studies.

The astonishing comparison between the outcomes of our RL studies leads to many open questions to be addressed in the future. It would be interesting to test (1) if harbor seals can improve their visual RL performance when location is used as conditional cue as was done in harp seals by Walsh et al. (2007), or (2) if harbor seals can successfully transfer the RL abilities acquired in a spatial task to a non-spatial RL task thus showing generalized learning such as chimpanzees, macaques (Macphail 1982) or corvids (Bond et al. 2007). Moreover, we think it could be promising to study harbor seals’ spatial, instead of visual (Gläser 2012; Mauck and Dehnhardt 2005; Scholtyssek et al. 2013), cognition in more detail in general.

In experiment 1, the two seals (1) completed R0 quickly, (2) the number of errors increased in R1, and (3) they gradually increased their performance over Rs. Leaving methodological differences, that most likely have an effect on RL performance (Mackintosh et al. 1985), for a moment aside, the number of errors during R0 as well as the rate of improvement is comparable to other mammals performing in RL tasks (see for example Doty and Combs 1969; Schusterman 1964).

Besides this overall similarity between the seals, a detailed analysis, however, revealed some performance differences between the experimental animals. Seal Moe (1) made significantly fewer errors than seal Sam, (2) reached the stage of one-trial learning, and (3) showed less fluctuation over Rs. When discussing these individual differences, it needs to be recalled that individual differences were also apparent in the visual RL experiment (Erdsack et al. this volume). In this past experiment, the performance of seal Sam was albeit superior to seal Moe’s; seal Sam mastered R0–R2 but failed during prolonged training in R3 whereas seal Moe already failed to complete R1. There are a number of factors that might generally account for individual differences, also in RL studies including other species (see for example Beach et al. 1974; Schusterman 1966), such as age, the character of the experimental animal, previous experience and motivation. The experimental animals of this study differ in all these aspects. We consider the first two aspects to have less explanatory value as these factors should have affected the spatial and visual RL experiment the same way, which is not applicable. The influence of the third aspect, previous experimental experience, is difficult to assess. Originally we would have expected that the broader experimental experience of seal Sam would cause his performance to be superior in comparison to seal Moe’s performance. Especially seal Sam’s slightly positive performance in the visual RL task (Erdsack et al. this volume) could have improved his performance in a later R task in line with Komischke (2013). However, transfer seems more effective with intradimensional changes than with extradimensional changes (Durlach and Mackintosh 1986), the latter holding for a change from visual to spatial RL. Seal Sam’s performance is indeed superior compared to its performance in the visual SRL experiment, but still inferior to seal Moe’s performance with seal Moe being generally less experienced. Thus, we cannot exclude that previous experience interfered with the current task. In our opinion, it is very probable that seal Sam performed inferior to seal Moe due to motivational factors. This notion is supported by seal Sam’s general behavior during experiments, meaning that he would sometimes take more time to start a session, to leave the hoop station and approach the targets than Moe, and his less stable performance over Rs such as for example his bad performance in R11.

The two harbor seals decreased their performance to an error rate lower than the error rate in R0, in R2 (seal Moe) and R4 (seal Sam) respectively. While seal Moe’s performance consistently stayed below the error rate in the original task, seal Sam’s performance fluctuated considerably, and his error rate was permanently below the error rate of R0 only from R12 onward. The fact that they achieved an error rate below the error rate during initial acquisition points to the fact that the seals were neither relearning in every R nor were storing both alternatives simultaneously. Instead they became more and more efficient which can be achieved by for example (1) learning a schedule or (2) learning set formation (Mackintosh et al. 1968). The first explanation can most likely be excluded as the seals kept on choosing the S+ from the previous day and did not autonomously switch to the new S+ in the final phase of the experiment when a R was completed every day; learning a schedule would have implied the anticipation of the new S+ with every new day/session. The second explanation implies that the animal learns to learn (Harlow 1949) and acquires rules according to which it can solve each new R more efficiently. As seal Moe was showing one-trial learning—in experiment 1 and 2—with a single error occurring at the beginning of a R, it possibly adopted a win-stay/lose-shift strategy (Levine 1959); this strategy was found to rule the response behavior of harbor seals in previous experiments (see for example Scholtyssek et al. 2013).

Further evidence for an underlying strategy—the strategy “choose left in the first trial of a session”—can be found in experiment 2 in which seal Moe was responding significantly more often on the left side in the first trial of an R. Interestingly, the most successful seal in the visual SRL study (Erdsack et al. this volume) had shown the same strategic behavior. Such a strategy is highly adaptive if the seal cannot remember the S+ of the previous session anymore. Thus, the response behavior of seals seems to be dominated by strategies which ultimately lead to an optimization of behavior; even in experiment 2, the seal was able to further improve its performance by increasing the number and frequency of single-error Rs. This result is remarkable as experiment 2 was started when the seal had reached asymptotic performance in experiment 1 with only 1–4 errors per R.

Experiment 2 revealed that a harbor seal was able to reverse its behavior several times within a session and reached the level of one-trial learning even under the more difficult/fluctuating experimental conditions. The seal often performed without further errors after a single error at the beginning of a R, which suggests that it used the recent history of reinforcement to guide its behavior in line with conditional discrimination (Williams 1971) or the application of a strategy, as previously discussed. A comparable behavior was documented in macaques, rats, and kea, in contrast to pigeons, in so-called mid-session Rs, in which a R occurs in the middle of the session (Laschober et al. 2021; Rayburn-Reeves et al. 2017, 2013); whereas pigeons seem to use temporal cues to anticipate the point of R and thus started choosing the alternative stimulus before the actual onset of the R; macaques, rats, and keas respond with a change in response behavior only after having experienced the first error/negative feedback. In contrast to the mentioned mid-session R studies, the current experimental design of Rs starting at variable points in time within the session did not allow the seal to use their well-developed timing abilities (Heinrich et al. 2016, 2020, 2021) to anticipate the point of R, but instead forced the seal to focus on the reinforcement history. In the future, the response strategy of harbor seals could be determined in a mid-session R to assess if the seals would still use the recent reinforcement history to control the response behavior or would anticipate the R based on temporal cues.

In experiment 3, with the help of a conditional cue, the harbor seal was able to perform errorless Rs. Our study contrasts with a previous conditional R learning study in harp seals (Walsh et al. 2007). The study by Walsh and coworkers required the seals to choose stimulus A in environment 1 and stimulus B in environment 2. Thus, the experimental animals needed to learn and memorize the association between the S+ and the environment. In our study, the environment stayed constant and instead an arbitrarily chosen external stimulus, the conditional stimulus, marked the beginning of a R, and thus a “change” of reward contingencies. However, the conditional stimulus did not carry the information about which stimulus was the S+. Taken together, the complexity of our experiment 3 seems to go far beyond associative learning. Experiment 3 required the seal to learn the meaning of the acoustic cue and moreover it was forced to keep the current S+ in its short-term memory; both challenges were mastered by the seal with ease, however, only in phase 2 of the experiment, when the conditional stimulus sounded after the start whistle. It remains to be determined if the seal learned the meaning of the conditional cue in phase 2 due to increased training effort, due to the changed timing or the changed salience of the stimulus. Altogether the seal’s performance in experiment 3 underlined its well-developed cognitive abilities and information processing capacity.

In conclusion, harbor seal individuals can master spatial RL experiments with increasing levels of difficulty. If generally present in harbor seals, the flexibility underlying this excellent performance is most likely highly advantageous regarding the optimization of various behaviors including for example foraging decisions. Spatial information in general and flexibility of handling spatial information could be required in the seals’ natural environment during trips from and to haul-out locations.

## Data Availability

All data are presented in the manuscript. Detailed learning performances can be obtained from the authors upon request.

## References

[CR1] Balderrama N (1980) One trial learning in the American cockroach, *Periplaneta americana*. J Insect Physiol 26:499–504 10.1016/0022-1910(80)90123-7

[CR2] Beach FA III, Pepper RL, Simmons JV Jr, Nachtigall PE, Siri PA (1974) Spatial habit reversal in two species of marine mammals. Psychol Rec 24:385–391 10.1007/BF03394257

[CR3] Bhumstein CT, Armitage KB (1998) Life history consequences of social complexity: a comparative study of ground-dwelling sciurids. Behav Ecol 9:8–19 10.1093/beheco/9.1.8

[CR4] Bodson A, Miersch L, Dehnhardt G (2007) Underwater localization of pure tones by harbor seals *(Phoca vitulina)*. J Acoust Soc Am 122:2263–2269 17902862 10.1121/1.2775424

[CR5] Bond AB, Kamil A, Balda RP (2007) Serial reversal learning and the evolution of behavioral flexibility in three species of North American corvids (*Gymnorhinus cyanocephalus*, *Nucifraga columbiana*, *Aphelocoma californica*). J Comp Psychol 121:372–379 18085920 10.1037/0735-7036.121.4.372

[CR7] Bullock DH, Bitterman ME (1962) Habit reversal in the pigeon. J Comp Physiol Psychol 55:958–962 14016793 10.1037/h0041070

[CR8] Bushnell PJ, Stanton ME (1991) Serial spatial reversal learning in rats: comparison of instrumental and automaintenance procedures. Physiol Behav 50:1145–1151 1798769 10.1016/0031-9384(91)90575-9

[CR56] Bublitz A, Dehnhardt G, Hanke FD (2021) Reversal of a spatial discrimination task in the common Octopus (*Octopus vulgaris*). Front Behav Neurosci. 10.3389/fnbeh.2021.614523 10.3389/fnbeh.2021.614523 PMC8267067 34248514

[CR57] Bublitz A, Weinhold SR, Strobel S, Dehnhardt G, Hanke FD (2017) Reconsideration of serial visual reversal learning in Octopus (*Octopus vulgaris*) from a methodological perspective. Front Physiol. 10.3389/fphys.2017.00054 10.3389/fphys.2017.00054 PMC5294351 28223940

[CR9] Chittka L (1998) Sensorimotor learning in bumblebees: long-term retention and reversal training. J Exp Biol 201:515–524 10.1242/jeb.201.4.515

[CR10] Davey G (1989) Ecological learning theory. Taylor & Frances/Routledge, Florence

[CR11] Day LB, Crews D, Wilczynski W (1999) Spatial and reversal learning in congeneric lizards with different foraging strategies. Anim Behav 57:393–407 10049480 10.1006/anbe.1998.1007

[CR12] Doty BA, Combs WC (1969) Reversal learning of object and positional discrimination by mink, ferrets and skunks. Q J Exp Psychol 21:58–62 10.1080/14640746908400195

[CR13] Dufort RH, Guttman N, Kimble GY (1954) One-trial discrimination reversal in the white rat. J Comp Physiol Psychol 47:248–249 13163264 10.1037/h0057856

[CR14] Durlach PJ, Mackintosh NJ (1986) Transfer of serial reversal learning in the pigeon. Q J Exp Psychol 38:81–95

[CR15] Easton A (2005) Behavioural flexibility, social learning, and the frontal cortex. The cognitive neuroscience of social behaviour. Psychology Press, New York, pp 59–79

[CR58] Erdsack N, Dehnhardt G, Hanke FD (accepted/this volume) Serial visual reversal learning in harbor seals (*(Phoca vitulina)*) Anim Cogn. 10.1007/s10071-022-01653-1 10.1007/s10071-022-01653-1 PMC9617845 35864326

[CR16] Fagan W et al (2013) Spatial memory and animal movement. Ecol Lett 16:1316–1329 23953128 10.1111/ele.12165

[CR17] Gläser N (2012) Visual and hydrodynamic orientation abilities of pinnipeds. University of Rostock

[CR18] Gonzalez RC, Behrend ER, Bitterman ME (1967) Reversal learning and forgetting in bird and fish. Science 158:519–521 6069100 10.1126/science.158.3800.519

[CR19] Hanke FD, Dehnhardt G (2009) Aerial visual acuity in harbor seals *(Phoca vitulina)* as a function of luminance. J Comp Physiol A 195:643–650 10.1007/s00359-009-0439-2 19360415

[CR20] Harlow HF (1949) The formation of learning sets. Psychol Rev 56:51–65 18124807 10.1037/h0062474

[CR21] Heinrich T, Dehnhardt G, Hanke FD (2016) Harbor seals *(Phoca vitulina)* are able to time precisely. Anim Cogn 19:1133–1142 27496205 10.1007/s10071-016-1020-3

[CR22] Heinrich T, Ravignani A, Hanke FD (2020) Visual timing abilities of a harbour seal *(Phoca vitulina)* and a South African fur seal *(Arctocephalus pusillus pusillus)* for sub- and supra-second time intervals. Anim Cogn 23:851–859 32388781 10.1007/s10071-020-01390-3 PMC7415748

[CR23] Heinrich T, Lappe A, Hanke FD (2021) Beyond the classic sensory systems: characteristics of the sense of time of harbor seals (*Phoca vitulina*) assessed in a visual temporal discrimination and a bisection task. Anat Rec. 10.1002/ar.24715 10.1002/ar.24715 34268905

[CR24] Holmes PA, Bitterman ME (1966) Spatial and visual habit reversal in the turtle. J Comp Physiol Psychol 62:828–831 10.1037/h0023675 6008016

[CR25] Jones CB (2005) Behavioral flexibility in primates: interpretations and prospects. Behavioral flexibility in primates: causes and consequences. Springer, Boston

[CR26] Komischke B, Giurfa M, Lachnit H, Malun D (2013) Successive olfactory reversal learning in honeybees. Learn Memory (cold Spring Harbor) 9:122–129 10.1101/lm.44602 PMC182587 12075000

[CR27] Krüger Y, Hanke W, Miersch L, Dehnhardt G (2018) Detection and direction discrimination of single vortext rings by harbour seals (*Phoca vitulina*). J Exp Biol. 10.1242/jeb.170753 29487151 10.1242/jeb.170753

[CR28] Kuroda T, Mozutani Y, Cancado CRX, Podlesnik CA (2017) Reversal learning and resurgence of operant behavior in zebrafish (*Danio rerio*). Behav Process 142:79–83 10.1016/j.beproc.2017.06.004 28633953

[CR29] Laschober M, Mundry R, Huber L, Schwing R (2021) Kea (*Nestor notabilis*) show flexibilty and individuality in within-session reversal learning tasks. Anim Cogn 24:1339–1351 34110523 10.1007/s10071-021-01524-1 PMC8492579

[CR30] Levine M (1959) A model of hypothesis behavior in discrimination learning set. Psychol Rev 66:353–366. 10.1037/h0044050 14416267 10.1037/h0044050

[CR31] Lucon-Xiccato T, Bisazza A (2017) Sex differences in spatial abilities and cognitive flexibility in the guppy. Anim Behav 123:53–60 10.1016/j.anbehav.2016.10.026

[CR32] Mackintosh NJ, Cauty A (1971) Spatial reversal learning in rats, pigeons, and goldfish. Psychon Sci 22:281–282 10.3758/BF03335956

[CR33] Mackintosh NJ, McGonigle B, Holgate V, Vanderver V (1968) Factors underlying improvement in serial reversal learning. Can J Exp Psychol 22:85–95 10.1037/h0082753 5649040

[CR34] Mackintosh NJ, Wilson B, Boakes RA (1985) Differences in mechanisms of intelligence among vertebrates. Philos Trans R Soc Biol Characters 308:53–65

[CR35] Macphail EM (1982) Brain and intelligence in vertebrates. Clarendon Press, Oxford

[CR36] Mauck B, Dehnhardt G (2005) Identity concept formation during visual multiple-choice matching in a harbor seal (*Phoca vitulina*). Learn Behav 33:428–436 16573213 10.3758/BF03193181

[CR37] Mauck B, Dehnhardt G (2007) Spatial multiple-choice matching in a harbour seal (*Phoca vitulina*): differential encoding of landscape versus local feature information? Anim Cogn 10:397–405 17377825 10.1007/s10071-007-0074-7

[CR38] Pavlov IP (1927) Conditional reflexes: an investigation of the physiological activity of the cerebral cortex. Oxford University Press, Oxford

[CR39] Ploog BO, Williams BA (2010) Serial discrimination reversal learning in pigeons as a function of intertrial interval and delay of reinforcement. Learn Behav 38:96–102 20065353 10.3758/LB.38.1.96

[CR40] Potter GD, Fiske JC (1979) Discrimination reversal learning in yearling horses. J Anim Sci 49:583–588 10.2527/jas1979.492583x

[CR41] Rayburn-Reeves RM, Stagner JP, Kirk CR, Zentall TR (2013) Reversal learning in rats (*Rattus norvegicus*) and pigeons (*Columba livia*): qualitative differences in behavioral flexibility. J Comp Psychol 127:205–211 10.1037/a0026311 22428983

[CR42] Rayburn-Reeves RM, James BT, Beran MJ (2017) Within-session reversal learning in rhesus macaque (*Macaca mulatta*). Anim Cogn 20:975–983 28755139 10.1007/s10071-017-1117-3

[CR43] Renouf D, Gaborko L (1989) Spatial and visual rule use by harbour seals (*Phoca vitulina*). Biol Behav 14:169–181

[CR44] Robinson MH (1985) Predator-prey-interactions, informational complexity, and the origins of intelligence. J Wash Acad Sci 75:91–104

[CR45] Scholtyssek C, Kelber A, Hanke FD, Dehnhardt G (2013) A harbor seal can transfer the same/different concept to new stimlus dimensions. Anim Cogn 16:915–925 23535852 10.1007/s10071-013-0624-0

[CR46] Schusterman RJ (1964) Successive discrimination-reversal training and multiple discrimination training in one-trial learning by chimpanzees. J Comp Physiol Psychol 58:153–156 14197033 10.1037/h0044309

[CR47] Schusterman RJ (1966) Serial discrimination-reversal learning with and without errors by the California sea lion. J Exp Anal Behav 9:593–600 5964516 10.1901/jeab.1966.9-593 PMC1338219

[CR48] Shettleworth SJ (1998) Cognition, evolution, and behavior. Oxford University Press, New York, Oxford

[CR49] Shultz S, Dunbar RIM (2006) Both social and ecological factors predict ungulate brain size. Proc R Soc B Biol Sci 273:207–215 10.1098/rspb.2005.3283 PMC1560022 16555789

[CR50] Tapp PD, Siwak CT, Estrada J, Head E, Muggenburg BA, Cotttman CW, Milgram NW (2003) Size and reversal learning in the beagle dog as a measure of executive function and inhibitory control in aging. Learn Memory (cold Spring Harbor) 10:64–73 10.1101/lm.54403 PMC196651 12551965

[CR51] Thompson PM, Miller D (1990) Summer foraging activity and movements of radio-tagged common seals (*Phoca vitulina*) in the Moray Firth Scotland. J Appl Ecol 27:492–501 10.2307/2404296

[CR52] Walsh SJ, Skinner DM, Martin GM (2007) Location serves as a conditional cue when harp seals (*Pagophilus groenlandicus*) solve object discrimination reversal problems. Can J Psychol 61:44–53 10.1037/cjep2007005 17479741

[CR53] Warren JM (1966) Reversal learning and the formation of learning sets by cats and rhesus monkeys. J Comp Physiol Psychol 61:421–428 4957108 10.1037/h0023269

[CR54] Weyant RG (1966) Reversal learning in rats as a function of the typ of discrimination and the criterion of learning. Anim Behav 14:480–484 5972807 10.1016/S0003-3472(66)80049-0

[CR55] Williams BA (1971) The effects of intertrial interval on discrimination reversal learning in the pigeon Psychonomic. Science 23:241–243

