# Correction to: Welldeveloped spatial reversal learning abilities in harbor seals (Phoca vitulina): Issue : 25_5_Issue

**DOI:** 10.1007/s10071-024-01893-3

**Published:** 2024-08-30

**Authors:** Benedikt Niesterok, Shanie Martin, Lisa Hildebrand, Guido Dehnhardt, Frederike D. Hanke

**Affiliations:** 1 Institute for Biosciences, Sensory and Cognitive Ecology, University of Rostock, Albert-Einstein-Str. 3, Rostock, 18059 Germany; 2 Faculty of Science and Engineering, Biological and Marine Science, University of Plymouth, Drake Circus, Plymouth, PL4 8AA UK; 3 Geospatial Ecology of Marine Megafauna Lab, Marine Mammal Institute, and Department of Fisheries, Wildlife and Conservation Sciences, Oregon State University, Newport, OR 97365 USA

In this article the Fig. 2 has been incorrectly published online.

The correct figure is given.

**Figure:**
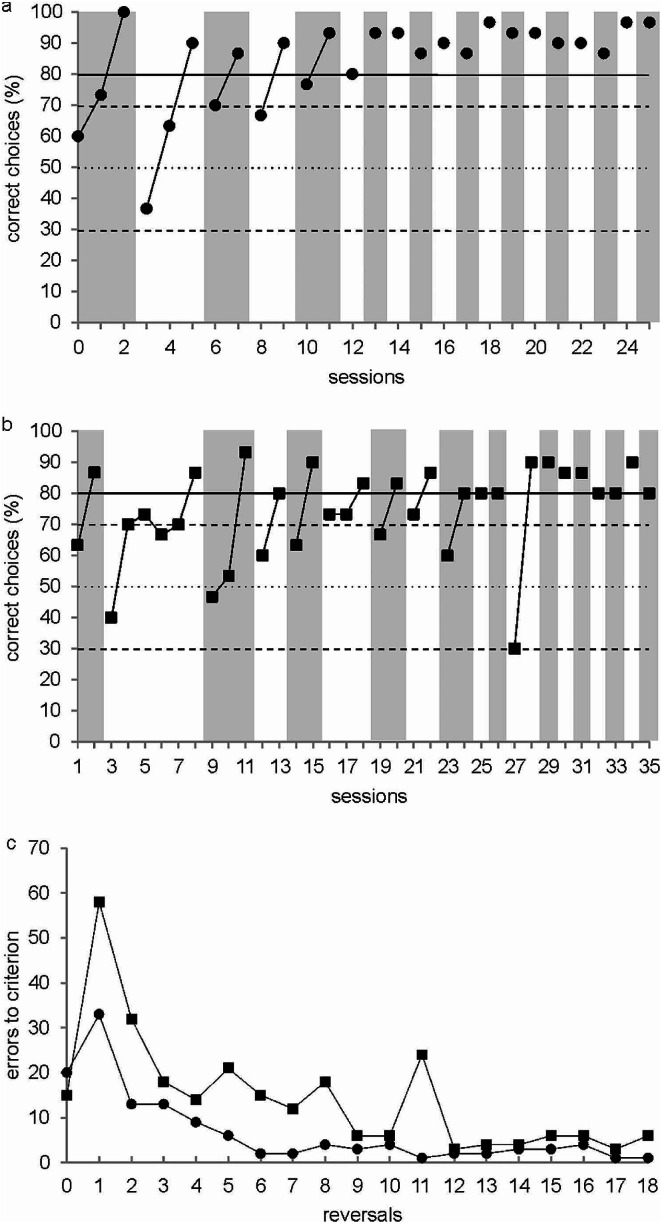

The Fig. 2 has been replaced in the original version of the article and published online.

